# Aggressive clinical course of epithelioid angiosarcoma in the femur: a case report

**DOI:** 10.1186/1477-7819-12-281

**Published:** 2014-09-11

**Authors:** Akio Sakamoto, Yusuke Takahashi, Yoshinao Oda, Yukihide Iwamoto

**Affiliations:** Department of Orthopaedic Surgery, Graduate School of Medical Sciences, Kyushu University, Fukuoka, 812-8582 Japan; Department of Anatomic Pathology, Graduate School of Medical Sciences, Kyushu University, Fukuoka, 812-8582 Japan

**Keywords:** Angiosarcoma, Bone, Epithelioid

## Abstract

**Background:**

Epithelioid angiosarcoma is a rare variant of angiosarcoma, and is characterized by an epithelioid morphologic appearance that mimics carcinoma. These tumors usually arise in extraskeletal sites; origination in bone is rare.

**Case presentation:**

A 69-year-old woman presented with right knee pain. Plain radiographs and magnetic resonance imaging showed an osteolytic lesion with a large soft-tissue extension into the distal femur. Under a diagnosis of metastatic carcinoma of unknown origin based on the biopsy specimen, resection and replacement with an artificial joint were performed. Histologic analysis of the resected material confirmed epithelioid angiosarcoma, supported by immunoexpression of cytokeratins and vascular markers. Three months after surgery, metastasis to the bone and lymph nodes was observed, and the patient died of the disease shortly thereafter.

**Discussion:**

Epithelioid angiosarcoma of bone is characterized by an aggressive clinical course. A possibility of epithelioid angiosarcoma of bone should be considered in cases with such epithelial features, particularly if only small specimens are available.

## Background

Angiosarcoma is a malignant mesenchymal neoplasm in which the tumor cells exhibit endothelial differentiation. Angiosarcoma frequently develops in sun-exposed skin of elderly individuals. It is characterized by aggressive biological behavior, resulting in a high rate of local recurrence, metastasis to lymph nodes, and systemic metastasis [1, 2].

Epithelioid angiosarcoma is a rare variant of angiosarcoma that is characterized by large cells with epithelioid features. It mimics poorly differentiated carcinoma through its epithelial appearance. Epithelioid angiosarcoma most often arises in the deep soft tissues of the extremities, although a variety of other primary sites, including the thyroid gland, skin, and adrenal glands, have also been reported [3, 4]. However, bone is a very rare origination site for this malignancy [5–7]. Herein, we report a case with epithelioid angiosarcoma in the bone characterized by an aggressive clinical course, in which the initial diagnosis on biopsy was metastatic carcinoma of unknown origin.

## Case presentation

**Figure 1:**
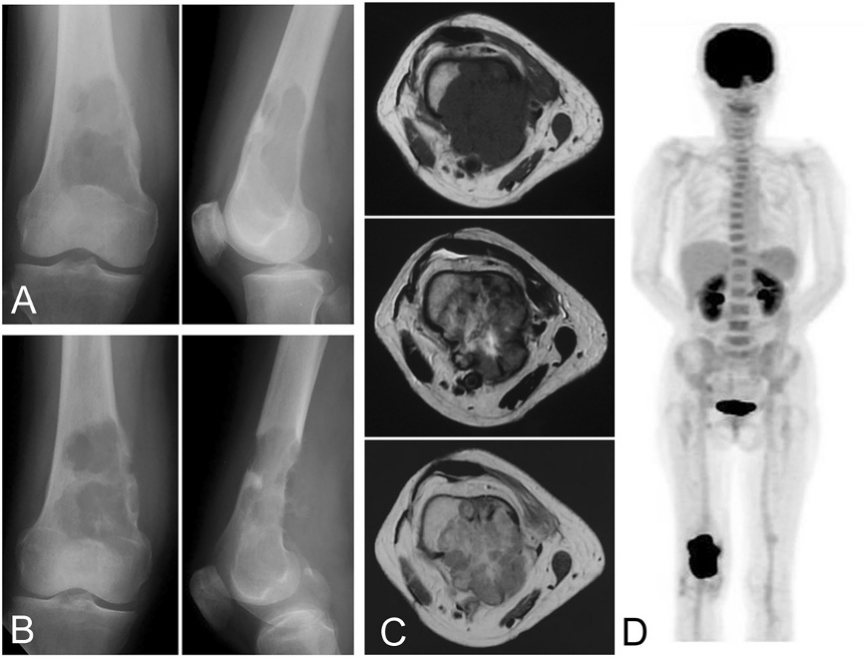
**Epithelioid angiosarcoma images. (A)** Plain X-radiographs show an osteolytic lesion in the distal femur. **(B)** This lesion has apparently widened after 1 month. **(C)** Magnetic resonance imaging shows a lobulated lesion with a low signal on T1-weighted imaging (top) and a heterogeneous signal on T2-weighted imaging (middle), and heterogeneous gadolinium enhancement is observed on T1-weighted imaging (bottom). **(D)** [^18^F]-2-fluoro-2-deoxy-D-glucose positron emission tomography reveals a solitary lesion in the distal femur.

A 69-year-old woman developed right knee pain 6 months prior to initial evaluation at our institute. She had tenderness over the distal thigh region, and the pain worsened on bearing weight. No laboratory data abnormalities were detected, except for an increased white blood cell count (15,000 white blood cells/μl) and elevation of the inflammatory marker C-reactive protein (5.27 mg/dl). Plain radiographs showed an osteolytic lesion with undefined margins and destroyed cortical bone in the distal femur. Neither periosteal reaction nor calcification was observed in the lesion (Figure 1A). Magnetic resonance imaging revealed that the lesion measured 7 cm in longitudinal diameter and was associated with a large soft-tissue mass that extended in a posterior direction. The lesions displayed low signal intensity on T1-weighted images and heterogeneous signal intensity on T2-weighted images. Gadolinium enhancement was observed heterogeneously on T1-weighted imaging (Figure 1C). Taking into consideration the patient’s age, the clinical diagnoses were metastatic carcinoma and primary malignant bone tumors consisting of leiomyosarcoma of bone, osteosarcoma, and undifferentiated high-grade pleomorphic sarcoma of bone.

**Figure 2:**
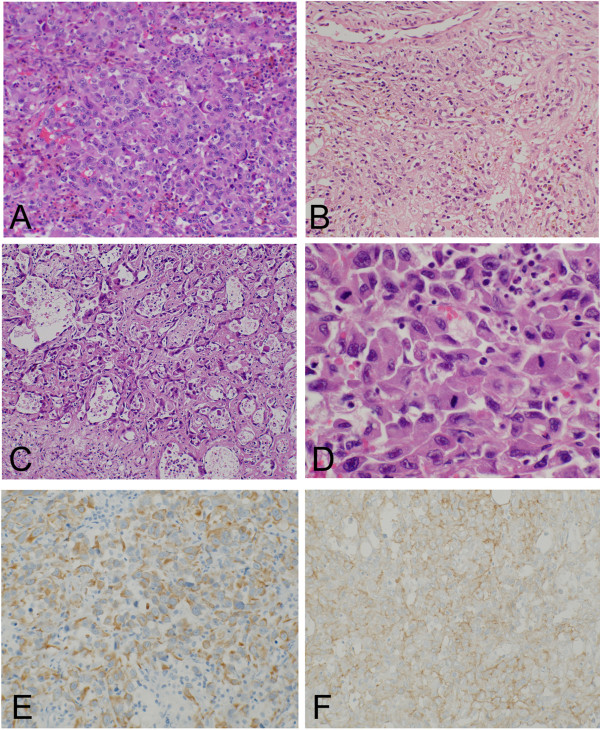
**Epithelioid angiosarcoma histology.** The tumor is composed of large, epithelioid cells with vesicular nuclei and prominent nucleoli **(A)**, with fibrous stroma **(B)**. **(C)** Vascular formation and malignant endothelial cells containing erythrocytes are observed. **(D)** Increased mitotic activity is observed. Immunohistochemically, tumor cells express cytokeratins AE1/AE3 **(E)** and the vascular marker CD31 **(F)**.

**Figure 3:**
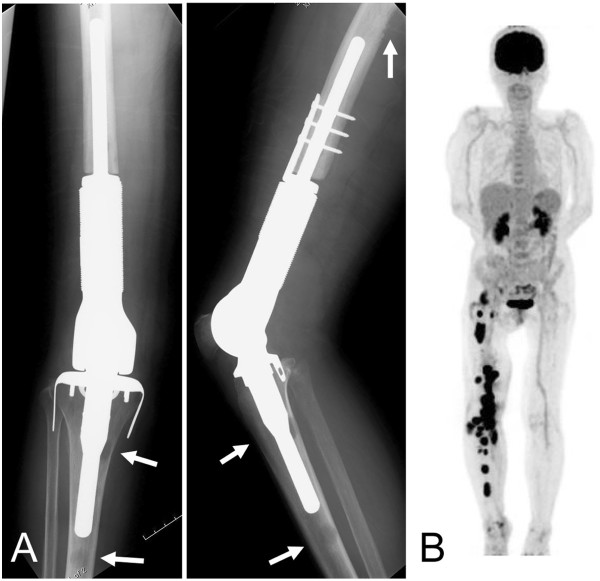
**Postoperative epithelioid angiosarcoma images. (A)** Three months after tumor resection and artificial joint replacement, plain radiographs show multiple osteolytic lesions at the femur and the tibia (arrows). **(B)** [^18^F]-2-fluoro-2-deoxy-D-glucose positron emission tomography shows multiple lesions extending from the pelvic cavity to the lower extremity.

A histological specimen on open biopsy suggested a diagnosis of metastatic carcinoma, owing to the epithelial morphology. To search for a primary carcinoma, computed tomography from neck to pelvis, gastrointestinal endoscopy, and colonoscopy were performed, but these failed to detect a lesion. Multiple tumor markers, including α-fetoprotein, carcinoembryonic antigen, CA125, CA19-9, and squamous cell carcinoma antigen were examined and their levels were found to be normal. No other lesions were identified by [^18^F]-2-fluoro-2-deoxy-D-glucose positron emission tomography (FDG-PET), in which the standardized uptake value was 12 (Figure 1D). The diagnosis of a solitary metastasis of unknown origin was made. Plain radiographs prior to resection, taken 1 month after the initial evaluation, showed that the area of lysis had widened and the cortex surrounding the lesion had disappeared (Figure 1B). Complete resection was performed, although the margin to the proximal major nerve and blood vessels was narrow. The bone was subsequently replaced with an artificial joint.Microscopically, the resected tumor cells appeared to have epithelioid features, and vascular channels or cystically dilated spaces were present. The tumor cells had abundant eosinophilic cytoplasm and large nuclei with prominent nucleoli (Figure 2). Immunohistochemically, the tumor cells expressed cytokeratins AE1/AE3 (Figure 2E) and CAM 5.2, as well as the vascular marker CD31 (Figure 2F). Expression of another vascular marker, CD34, was not detected, and factor VIII expression was faint.Three months after surgery, plain radiographs showed multiple osteolytic lesions at the femur and tibia along the artificial joint (Figure 3A), while FDG-PET showed multiple metastases, distributed from the right pelvis to the leg (Figure 3B). The patient died of the disease shortly thereafter.

### Discussion

Epithelioid angiosarcoma is a variant of angiosarcoma, which is composed of neoplastic cells with an epithelioid morphologic appearance. This type of tumor is characterized by poor differentiation and biological aggressiveness. A previous series of ten cases of epithelioid angiosarcoma of bone is summarized in Table 1. The series included eight men and two women, who ranged in age from 26 to 83 years (mean, 62 years) [7]. The current patient is 69 years old, which is consistent with this age range. Of the previous ten cases, four were solitary and six were multifocal. Of the four solitary tumors, three were located in the femur [7]. The initial tumor location in the current case was also the femur; thus, the femur appears to be a frequent site of solitary metastasis.

**Table 1:** **Clinical summary of reported ten cases with epithelioid angiosarcoma in the bone**

Mean age (range)	Sex (male/female)	Solitary (4/10; 40%)	Multifocal (6/10; 60%)	Metastasis 7/9 (78%)	Died of disease 7/9 (78%)
62 years old (26 to 83)	8/2	Reported sites in more than one case			Time to death
		Femur	Femur	Lung	7 weeks to 27 months
			Tibia	Soft tissue	
			Pelvis	Bone	
		Reported sites in one case			Time to death (<2 years)
		Calcaneus	Scapula	Lymph node	6/7 (86%)
			Radius		
			Carpal bone		
			Metacarpal bone		
			Rib		
			Lumbar vertebra		

The table is modified from Table one in reference [7].

Metastatic carcinoma can be difficult to distinguish from epithelioid angiosarcoma. Both tumors are composed of epithelioid tumor cells, and they tend to affect older individuals. In a previous report, 3 of 10 cases were misdiagnosed as metastatic carcinoma [7]. Furthermore, the cytokeratin expression characteristic of epithelioid angiosarcoma may lead to a misdiagnosis of metastatic carcinoma. Identifying histologic features of epithelioid angiosarcomas, including the presence of well-formed vascular channels and cytoplasmic vacuoles that contain red blood cell fragments, is essential. However, the small amount of sample that can be obtained with biopsy may also lead to an incorrect diagnosis of metastatic carcinoma. Epithelioid hemangioendothelioma is also a differential diagnosis of epithelioid angiosarcoma, because both tumors share a number of histopathologic features of epithelioid cells with intracytoplasmic lumina. However, epithelioid hemangioendothelioma is a low-grade malignant vascular tumor with minimal cellular pleomorphism. Epithelioid hemangioendothelioma is less aggressive than epithelioid angiosarcoma [7, 8].

Vascular marker expression patterns are useful for the diagnosis of epithelioid angiosarcoma. Among vascular markers, CD31 has been reported to be the most sensitive [7]. However, it is important to be aware that expression of all vascular markers does not have to be negative. In the current case, factor VIII was faintly expressed, while CD34 expression was negative. In a previous report, factor VIII immunoexpression was positive in 6 of 8 cases, and CD34 expression was detected in only 2 of 5 cases [7].

Epithelioid angiosarcoma can metastasize to both lymph nodes and organs, including the lungs, bone, soft tissue, and skin, whereas most malignant soft-tissue tumors only metastasize to the lungs. More than 50% of patients with epithelioid angiosarcomas die from the disease within 2 to 3 years of diagnosis [2, 3, 7]. In the current case, the tumor recurred 3 months after surgery, and the patient died shortly thereafter, owing to quick spread of the cancer. Bone metastasis was not only proximal to, but also at a site from the original bone lesion. FDG-PET was useful for detecting multiple metastases to lymph nodes and bones.

## Conclusions

In summary, we have reported that epithelioid angiosarcoma of the bone is characterized by an aggressive nature. To differentiate it from a metastatic tumor, careful histological examination using immunoexpression of vascular markers is critical. The diagnosis should be included in the differential diagnosis of epithelioid neoplasms of the bone, particularly for small specimens.

## Consent

The patient and the families were informed that data from the case would be submitted for publication, and provided their consent accordingly.

## Abbreviations

FDG-PET: [^18^F]-2-fluoro-2-deoxy-D-glucose positron emission tomography.
